# Pathogenicity study in sheep using reverse-genetics-based reassortant bluetongue viruses

**DOI:** 10.1016/j.vetmic.2014.09.012

**Published:** 2014-11-07

**Authors:** Cristina C. Celma, Bishnupriya Bhattacharya, Michael Eschbaumer, Kerstin Wernike, Martin Beer, Polly Roy

**Affiliations:** a Department of Pathogen Molecular Biology, Faculty of Infectious and Tropical Diseases, London School of Hygiene and Tropical Medicine, United Kingdom; b Institut für Virusdiagnostik, Friedrich-Loeffler-Institut, Greifswald-Insel Riems, Germany

**Keywords:** Bluetongue virus, Serotype 8, Reassortment, Non-structural protein NS3, Pathogenicity in sheep

## Abstract

Bluetongue (BT) disease, caused by the non-enveloped bluetongue virus (BTV) belonging to the *Reoviridae* family, is an economically important disease that affects a wide range of wild and domestic ruminants. Currently, 26 different serotypes of BTV are recognized in the world, of which BTV-8 has been found to exhibit one of the most virulent manifestations of BT disease in livestock. In recent years incursions of BTV-8 in Europe have resulted in significant morbidity and mortality not only in sheep but also in cattle. The molecular and genetic basis of BTV-8 pathogenesis is not known. To understand the genetic basis of BTV-8 pathogenicity, we generated reassortant viruses by replacing the 3 most variable genes, S2, S6 and S10 of a recent isolate of BTV-8, in different combinations into the backbone of an attenuated strain of BTV-1. The growth profiles of these reassortant viruses were then analyzed in two different ovine cell lines derived from different organs, kidney and thymus. Distinct patterns for each reassortant virus in these two cell lines were observed. To determine the pathogenicity of these reassortant viruses, groups of BTV-susceptible sheep were infected with each of these viruses. The data suggested that the clinical manifestations of these two different serotypes, BTV-1 and BTV-8, were slightly distinct and BTV-1, when comprising all 3 genome segments of BTV-8, behaved differently to BTV-1. Our results also suggested that the molecular basis of BT disease is highly complex.

## Highlights

• Use of reverse genetics to generate reassortant BTV viruses for testing in animals.
• Two structural and one non-structural proteins are involved in pathogenicity.
• Molecular basis of bluetongue disease appears to be highly complex.

## 1 Introduction

Bluetongue (BT), an insect-transmitted, non-contagious viral disease of domestic and wild ruminants is caused by bluetongue virus (BTV). The disease is characterized by inflammation of the mucous membranes, congestion, swelling, hemorrhages and is often accompanied with high mortality in sheep (Erasmus, 1975, Maclachlan et al., 2009). Although cattle and goats usually carry the virus for a certain period of time without showing any apparent clinical signs of disease, they are capable of transmitting the virus to other ruminants via biting *Culicoides* midges. BTV is present in a broad band of countries extending approximately between 40°N and 35°S (Mellor et al., 2000, Purse et al., 2005). Until 15 years ago, Europe was essentially BT-free apart from Cyprus; however, since 1998 at least one of the 26 serotypes of BTV has been active on the continent every year, mainly in the Mediterranean basin (Maclachlan and Guthrie, 2010, Mellor et al., 2008, Purse et al., 2005). In 2006 a highly pathogenic BTV-8 strain emerged for the first time in Northern Europe, spreading very rapidly and affecting thousands of herds. The same serotype re-emerged in 2007 and 2008, causing devastating disease not only in sheep but also in cattle with high morbidity and mortality (Elbers et al., 2008a, Elbers et al., 2008b, Wilson and Mellor, 2009). Studies involving molecular epidemiology have also shown that the most severe disease in northern European sheep and cattle was caused by BTV-8 (Dal Pozzo et al., 2013, Martinelle et al., 2013, Purse et al., 2005). The phenotypic differences between BTV-8 compared with less virulent strains suggested that genetic background may be partly responsible. However, the mechanism of pathogenicity is still very poorly understood.

BTV is a member of the *Orbivirus* genus within the *Reoviridae* family. Like other members of the family, BTV has a genome of 10 segmented double-stranded RNA (segments S1–S10) that are enclosed within two capsids. While the inner core is made up of 5 highly conserved proteins (VP1, VP3, VP4, VP6 and VP7), the outer capsid consists of two variable proteins, VP2 (receptor-binding protein and serotype determinant) and VP5 (membrane penetration protein). In addition, BTV also encodes for 4 non-structural proteins (NS1–NS4), of which NS3 encoded by S10 is more variable than NS1 and NS2. NS3 is shown to be involved in virus trafficking and release from the infected host (Beaton et al., 2002, Celma and Roy, 2009). Recently it has been shown that NS3 is also involved in the regulation of the induction of interferon type 1 (Chauveau et al., 2013), suggesting a role in the innate immune response.

In this study, we designed reassortant viruses between BTV-8 and BTV-1 to establish the genetic basis of BTV pathogenicity. The rationale for designing reassortant viruses was based on the two most variable proteins of the outer capsid (VP2 and VP5) and the non-structural protein NS3, which is the most variable within BTV NS proteins. Reassortant viruses were generated using a reverse genetics (RG) system replacing these three RNA segments (S2, S6 and S10) of the low virulent strain, BTV-1, with that of highly virulent BTV-8, either singly or in combinations. The phenotypic characteristics of the disease caused by these reassortant viruses were analyzed by infection of sheep. Our results suggested that all three proteins together are involved in the disease outcome and that the molecular basis of BTV pathogenicity is highly complex.

## 2 Methods

### 2.1 Cells and viruses

BSR cells (BHK-21 subclone) were maintained in Dulbecco modified eagle medium (DMEM, Sigma–Aldrich) supplemented with 5% (v/v) fetal bovine serum (FBS, Invitrogen). PT and SFT-R cells (ovine-derived kidney and thymus cells respectively, Collection of Cell Lines in Veterinary Medicine, Friedrich-Loeffler-Institut, Insel Riems, Germany) were maintained in minimum essential medium eagle (MEM, Sigma–Aldrich) supplemented with 10% (v/v) FBS.

BTV-1 (South African strain) and BTV-8 (Ardennes isolate) viral stocks were generated by infection of BSR cells and kept at 4 °C until use.

### 2.2 Recovery of reassortant BTV-1/BTV-8 viruses

Segments S2 (VP2), S6 (VP5) and S10 (NS3) (GenBank accession numbers: KJ872780–KJ872782) of BTV-8 were obtained using a sequence-independent cloning system as previously described (Boyce et al., 2008, Maan et al., 2007, Matsuo et al., 2011). Briefly, dsRNAs from purified core particles were ligated to a self-annealing primer before RT-PCR amplification using a specific primer. Each cDNA amplified from segments S2, S6 and S10 of BTV-8 was cloned into pUC19 and fully sequenced (Source Bioscience) before insertion of the T7 promoter at the 5′ end and insertion of a unique restriction enzyme site that generates the correct end of the segment at the 3′ end.

For synthesis of uncapped T7 transcripts for segments S2, S6 and S10, RiboMAX Large-Scale RNA Production System T7 (Promega) or TranscriptAid T7 High Yield Transcription Kit (Thermo Scientific) kits were used according to manufacturer's instructions. Reassortant viruses between BTV-1 and BTV-8 were recovered from confluent monolayers of BSR cells after transfection with a full set of BTV T7 transcripts as described before (Boyce et al., 2008). Individual plaques were picked, amplified and virus stocks were kept at 4 °C.

### 2.3 Virus growth kinetics and characterization

The genomic dsRNA profile was analyzed for each reassortant. Monolayers of BSR cells were infected with reassortant or parental viruses and upon complete cytopathic effect, infected cells were harvested. The genomic dsRNA was purified with Tri reagent (Sigma) using standard methods. The parental origin of segments S2, S6 and S10 was determined by differential mobility in non-denaturing PAGE or by sequencing.

For virus growth study, monolayers of PT and SFT-R cells were synchronously infected at a multiplicity of infection (MOI) of 1 and samples were collected at 0, 12, 24 and 48 h (hours) post-infection (p.i.). Cells and supernatant were harvested, subjected to two freeze/thaw cycles and the total titer was determined by plaque assay in triplicate and expressed as plaque formation units per ml (PFU/ml) or by tissue culture infective dose 50 (TCID_50_). The mean, standard deviation and the *p* values were also determined by Excel (Microsoft). Viral protein expression was determined by Western blot (WB) using specific antibodies against structural VP5, VP7 and non-structural NS3 proteins. As loading control, an antibody against β-actin (Sigma) was used. Each blotting experiment was repeated twice and the amount of protein expression was quantitated by ImageJ software.

### 2.4 Pathogenicity studies

Forty-six sheep (East Frisian breed) were segregated in groups of 4 animals and subcutaneously injected with parental BTV-1 (South African strain), BTV-8 (Ardennes isolate) or one of the reassortant viruses at ∼1 × 10^7^ PFU/animal. Control groups received only saline buffer. Whole blood samples were taken at regular intervals before and after infection. For three weeks, a clinical score was assigned daily to each sheep based on a range of clinical signs. These included body temperature, feed intake, attitude, and lameness, appearance of conjunctiva, scleral blood vessels and cornea, appearance of skin and mucous membranes of the nose and mouth, redness and hemorrhaging, nasal and ocular discharge, salivation, respiratory rate and sounds, as well as edema of the tongue, face, ears, periorbital and submandibular regions. Depending on severity, up to four points were awarded per category. All animal procedures were in compliance with European ethical regulations, i.e. directives 91/628, 92/65 and 86/609/EEG, regarding the protection of vertebrate animals used for experimental and/or scientific purposes.

A mixed-design analysis of the variance model (implemented in R, http://www.r-project.org/) was used to test for significant differences in clinical scores between groups. *P* values from post hoc pairwise comparisons were adjusted for multiplicity by the Bonferroni method.

### 2.5 Viral load

To determine virus replication in animal hosts, RNA was extracted from blood samples at different time points with the NucleoSpin 96 virus core kit (Macherey-Nagel) and analyzed by real-time RT-PCR as described previously (Toussaint et al., 2007) using a LightCycler 480II (Roche Diagnostics). A NS1 (S5) RNA standard was used for absolute quantification.

### 2.6 Immunological tests

The presence of antibodies against BTV VP7 was measured by ELISA. Serum samples were analyzed by a double-recognition BTV antibody ELISA (PrioCHECK BTV DR, Prionics GmbH). For neutralization tests, serum samples were tested against parental BTV-1 and BTV-8 viruses and the neutralization titer was determined as the highest dilution able to neutralize 100 TCID_50_ of virus in 50% of replicate wells.

## 3 Results

### 3.1 Recovery and characterization of reassortant BTV-1 and BTV-8 viruses

Initially BTV-8 (Ardennes isolate) (Le Gal et al., 2008) exact-copy segments S2, S6 and S10, encoding VP2, VP5 and NS3 respectively, were cloned using a sequence-independent method (Boyce et al., 2008, Maan et al., 2007). Subsequently, after ensuring the accuracy of the clones by sequencing, a T7 promoter and a specific restriction enzyme site were introduced by PCR and transcripts were generated using the fully digested plasmids as templates. For virus recovery, BSR cells were transfected with ssRNAs S2, S6 and S10 of BTV-8 in different combinations together with the remaining ssRNAs (either 7 or 8 segments) of BTV-1 (Boyce et al., 2008). Following incubation, reassortant viruses were recovered as described previously (Boyce et al., 2008) and assessment of the plaque morphology established that the recovered BTV1/8VP2.5 (with segments S2 and S6 of BTV-8) and BTV1/8VP2.5.NS3 (with segments S2, S6 and S10 of BTV-8) reassortant viruses had plaque formation similar to the parental viruses (data not shown).

**Fig. 1:**
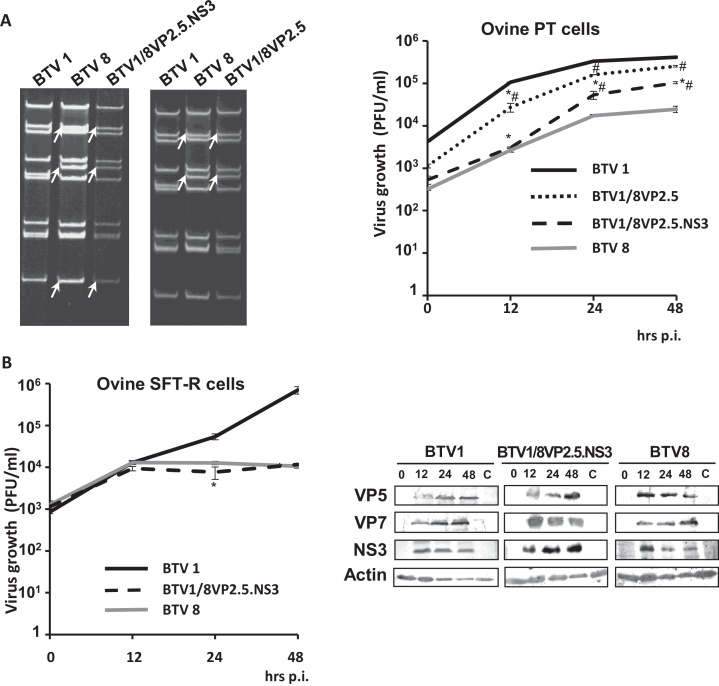
Characterization of reassortant BTV-1/BTV-8 viruses. (A) Left panel, genomic profile of reassortant viruses generated by RG. DsRNA was extracted from cells infected with either BTV-1, BTV-8, BTV1/8VP2.5.NS3 or BTV1/8VP2.5 and analyzed in a non-denaturing polyacrylamide gel. Arrows indicate reassortant segments S2, S6 or S10 of BTV-8 in each generated virus. Right panel, virus growth profile in the ovine kidney-derived PT cell line. Cells were infected with parental BTV-1 or BTV-8, or reassortant BTV1/8VP2.5.NS3 or BTV1/8VP2.5 and samples were harvested at the indicated times. Titers at each time point were determined in triplicate by plaque assay, expressed as virus growth in plaque formation units per ml (PFU/ml) and plotted in logarithmic scale. Error bars indicate standard deviation. Asterisk and hashtag indicates the significance (*p* < 0.05) of the difference in titers of BTV1/8VP2.5.NS3 or BTV1/8VP2.5 at 12, 24 and 48 h p.i. to WT BTV-1 (*) or BTV-8 (#). (B) Characterization of BTV1/8VP2.5.NS3 in ovine thymus-derived SFT-R cells. Left panel, virus growth. Cells were infected with BTV1/8VP2.5.NS3 or parental BTV-1 or BTV-8 and processed as indicated in (A). The *p* values were designated the same way as in (A). Right panel, protein expression profile from cells infected with BTV1/8VP2.5.NS3 at the indicated time points. Viral proteins were detected by Western blot using specific antibodies against VP5, VP7 or NS3. As loading control, an antibody against cellular actin was used. Protein profile from cells infected with BTV-1 and BTV-8 was used as controls.

Purification of the genomic dsRNAs from cells infected with independent plaques of BTV1/8VP2.5 and BTV1/8VP2.5.NS3 and their subsequent analysis on a non-denaturing polyacrylamide gel confirmed that the recovered reassortant viruses had the expected dsRNA genome patterns that were equivalent to the parental origin of the segments (Fig. 1A, left panel). In addition, RT-PCR and sequencing also validated the authenticity of the segments that could not be assessed by their mobility in non-denaturing polyacrylamide gels.

Since pathogenicity of BT disease is more pronounced in sheep, ovine cells were evaluated for growth kinetics and protein expression at different times p.i. For this purpose, first an ovine cell line (PT) derived from kidney was infected at MOI of 1 with either wild-type BTV-1 or BTV-8 or reassortant virus BTV-1/8VP2.5 or BTV-1/8VP2.5.NS3. Virus growth kinetics was monitored by plaque assay (Fig. 1A, right panel) of samples taken at 0, 12, 24 and 48 h p.i. Although both reassortant viruses BTV1/8VP2.5 and BTV1/8VP2.5.NS3 consisted of a BTV-1 backbone, the growth characteristics of only the triple reassortant were different to that of BTV-1. Reassortant virus BTV1/8VP2.5 exhibited a similar profile to BTV-1, with only a slight difference at 12 h (*p* < 0.05) but not at (*p* > 0.05) either 24 or 48 h p.i. In contrast, the triple reassortant BTV-1/8VP2.5.NS3 virus titer at 12 h p.i. was significantly different from BTV-1 (*p* < 0.05) but not from BTV-8 at the same time point (*p* > 0.05). However, growth of this virus at later times (24 and 48 h p.i.) exhibited a somewhat different (*p* < 0.05) profile in comparison to both parental viruses, BTV-1 and BTV-8, albeit closer to BTV-8.

Since triple reassortant virus BTV1/8VP2.5.NS3 appeared to have a different growth profile to that of BTV-1, the growth patterns of these two viruses were further analyzed using an alternate ovine cell line, ovine thymus SFT-R cells. As VP2, VP5 and NS3 in the triple reassortant virus BTV1/8VP2.5.NS3 were taken from BTV-8, the latter was also included as a control. Analysis of the growth profiles of the three viruses in SFT-R cells (Fig. 1B, left panel) exhibited that the virus titers of the reassortant virus, BTV-1 and BTV-8 at 12 h p.i. were similar and there was no statistically significant difference (*p* > 0.05) between them. Although virus titers of reassortant and BTV-8 plateaued by 24 h p.i., the only virus that still continued to grow at the later time points was the BTV-1. Further, Western blot analysis of viral protein expression, using antibodies for NS3, VP5 and a major structural protein VP7 (Fig. 1B, right panel), revealed that all three viruses expressed these proteins as early as 12 h p.i. Since the virus titers of the triple reassortant virus, BTV-1 and BTV-8 were not statistically significant from each other at 12 h p.i., the protein expression for all three viruses was measured at 24 and 48 h p.i. Further, densitometric analysis of protein expression at each time point indicated that the increase in expression of VP7 and VP5, but not NS3, was similar to BTV-8; while NS3 was more similar to BTV-1 (results not shown). Altogether, this suggested that exchanging S2, S6 and S10 segments of BTV-1 with that of BTV-8 resulted in a reassortant virus that was more similar to BTV-8 than BTV-1. In addition, the effect of the reassortment was more pronounced in the thymus-derived (SFT-R) than in the kidney (PT) cells.

### 3.2 Pathogenicity in sheep infected with BTV reassortant viruses

**Fig. 2:**
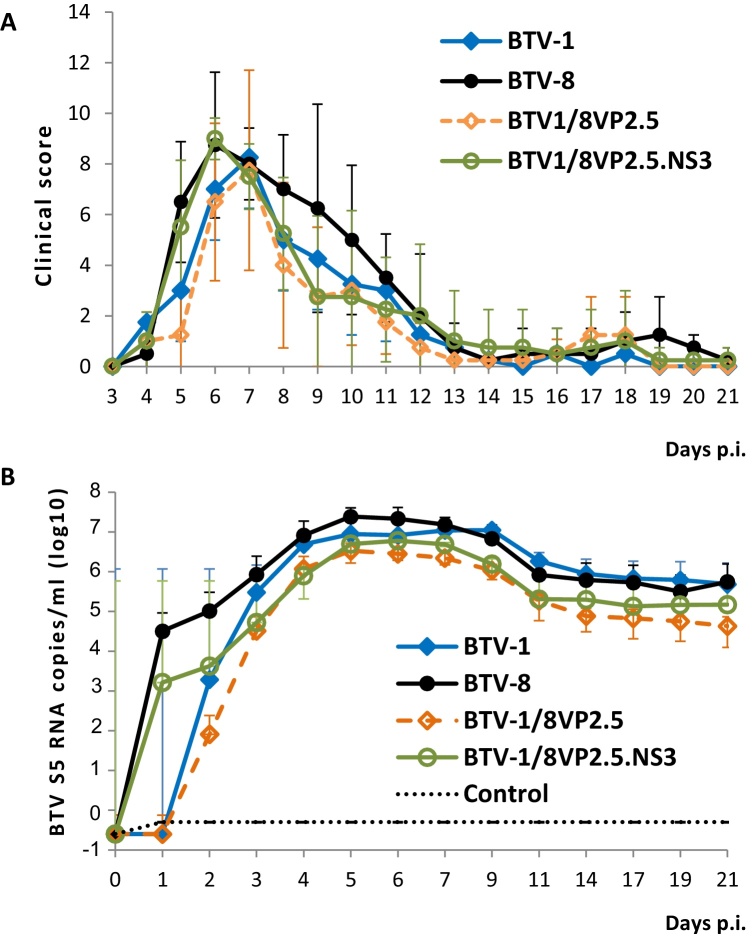
Clinical manifestation and virus replication in sheep infected with reassortant BTV1/BTV-8 viruses. (A) Animals were inoculated with reassortants BTV1/8VP2.5.NS3, BTV1/8VP2.5 or parental BTV-1 or BTV-8. Infected animals were examined daily. Attitude, body temperature and other clinical signs were recorded and scored as outlined in the text. Means and standard deviation were calculated for each group. (B) Animals were inoculated with virus as above and blood samples for detection of NS1 gene by real-time RT-PCR were taken at the indicated time points. As control, a group of animals injected with saline buffer was included in the study.

Since in tissue culture the triple reassortant virus BTV1/8VP2.5.NS3 behaved more similarly to BTV-8 and BTV-1/8VP2.5 to BTV-1, experiments were designed to analyze the pathogenicity of these reassortant viruses in animal hosts. For this purpose, four groups containing four sheep in each group were infected with BTV-1 or BTV-8 or BTV1/8VP2.5 or BTV1/8VP2.5.NS3. Two animals injected with saline buffer were used as the control group. The pathogenicity of each virus infection was analyzed by clinical observation of the animals in all groups. A clinical score was assigned daily to each sheep based on a range of clinical symptoms as described in Section 2. Depending on severity, up to four points were awarded per category. Animals infected with the reassortant BTV1/8VP2.5.NS3 presented a slightly early onset of the highest clinical score compared to BTV1/8VP2.5 or BTV-1 (Fig. 2A). As the same pattern was also observed for BTV-8 with a maximum clinical score at 6 days p.i., this data suggested that the NS3 might influence virus pathogenicity and might play an important role in the onset of BT disease. Although the clinical scores in all four infection groups were different from the control group, the observed differences between the infection groups were not statistically significant.

Subsequently, viremia in these infected animals was estimated by analyzing the whole blood samples that were taken before virus inoculation and on days 1 to 7, 9, 11, 14, 17, 19, and 21 p.i. using real-time RT-PCR (Toussaint et al., 2007). Animals infected with the reassortant viruses showed an increase in the number of NS1 gene copies over the first week after infection (Fig. 2B) similar to BTV-1 and BTV-8 viruses, indicating that these viruses can replicate as efficiently as parental viruses in an animal host. In comparison, samples from control sheep were free of BTV RNA. (Fig. 2B, control). While productive infection measured by detecting the presence of antibodies against VP7, the group-specific antigen, by ELISA, also demonstrated that all animals in the infection groups had seroconverted by day 7 after inoculation, no BTV antibody response was detected in any sample from the negative control sheep (data not shown). In addition, the serum neutralization response tested in samples taken at 21 days p.i. confirmed that animals infected with the reassortant viruses BTV1/8VP2.5.NS3 and BTV1/8VP2.5 showed a strong neutralization response against BTV-8 but not BTV-1 (Table 1). In comparison, animals infected with BTV-1 presented a response only against the same serotype. Overall these data indicated that the reassortant viruses are able to replicate in animals and they also generate a strong immune response similar to that of the parental strains. Since the sheep infected with the reassortant BTV1/8VP2.5.NS3 showed a clinical score that was comparable to that of BTV-8, it was hypothesized that NS3 might be playing an important role in the pathogenicity of BT disease.

**Table 1:** Serum neutralization titer in animals infected with BTV1/8VP2.5.NS3, BTV1/8VP2.5 or parental BTV-1 or BTV-8.

Infection group	BTV-1 (dilution)^a^	BTV-8 (dilution)^a^
BTV-1 (4 animals)	1/640	Negative
	1/240	Negative
	1/240	Negative
	1/480	Negative

BTV-8 (4 animals)	Negative	1/480
	Negative	1/240
	Negative	1/240
	Negative	1/320

BTV1/8VP2.5 (4 animals)	Negative	1/240
	Negative	1/240
	Negative	1/120
	Negative	1/120

BTV1/8VP2.5.NS3 (4 animals)	Negative	1/480
	Negative	1/320
	Negative	1/480
	Negative	1/120

PBS (2 animals)	Negative	Negative
	Negative	Negative

Negative indicates no detectable neutralizing activity in a 1/10 dilution.

a Neutralizing activity in serum samples from sheep 21 days post-infection with reassortant or parental virus was determined as the highest dilution that neutralized 100 TCID_50_ virus in 50% of replicate wells.

### 3.3 Importance of NS3 in bluetongue disease in sheep

**Fig. 3:**
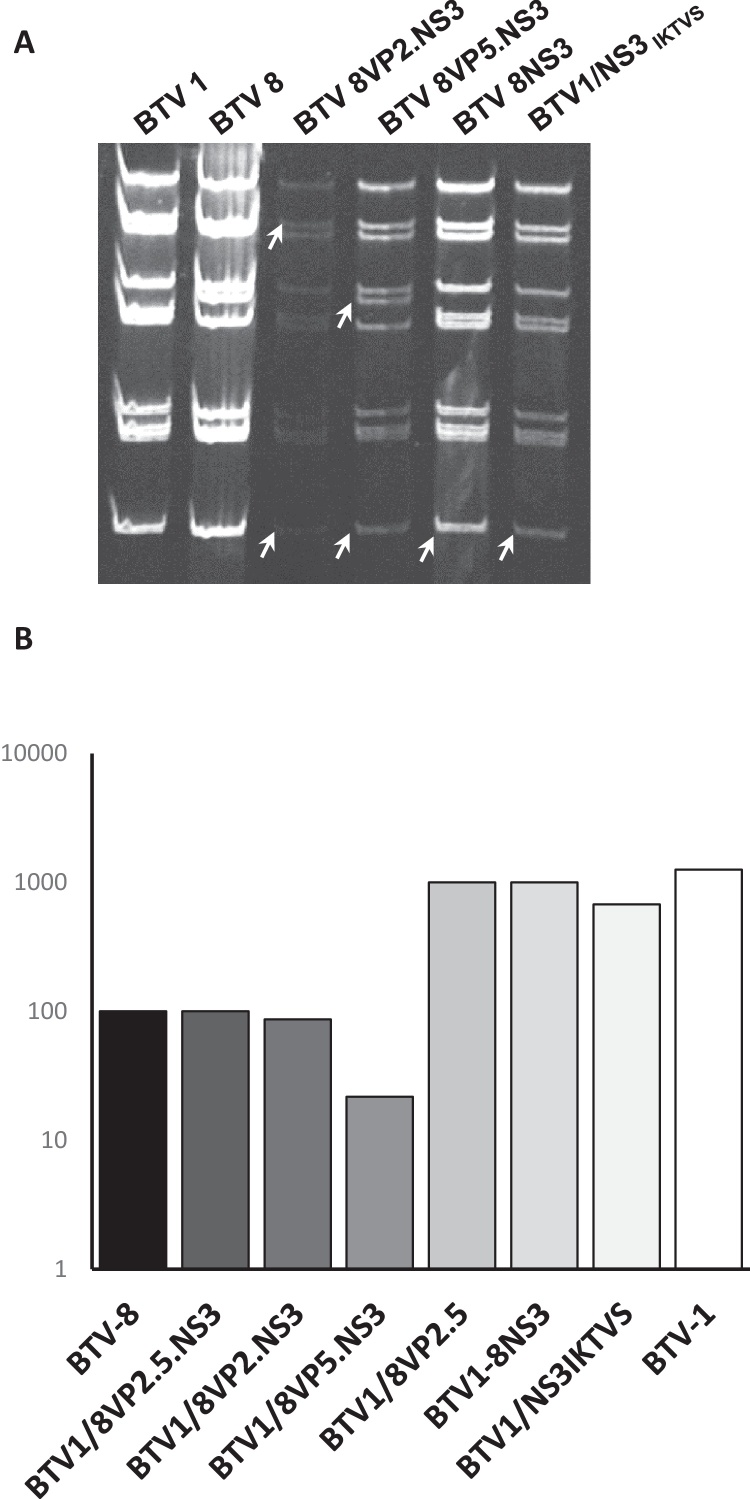
Characterization of reassortants BTV1/8VP2.NS3, BTV1/8VP5.NS3, BTV1/8NS3 or mutant BTV1/NS3_IKTVS_ viruses. (A) Genomic profile of reassortant viruses generated by reverse genetics. Genomic dsRNA was extracted from cells infected with either reassortant or parental viruses as indicated in each lane and analyzed in a non-denaturing polyacrylamide gel. (B) Virus growth in SFT-R cell line. Cells were infected with parental BTV-1 or BTV-8 or triple reassortant BTV1/8VP2.5.NS3 or dual reassortant BTV1/8VP2.NS3, BTV1/8VP5.NS3 and BTV1/8VP2.5 or single BTV1/8NS3 and BTV1/NS3_IKTVS_ viruses and samples were harvested at 48 h p.i. Total titers were determined by TCID_50_ and expressed as percentage of BTV-8 titer considered as 100%.

To further determine the importance of NS3 in pathogenicity, we generated a reassortant virus in which S10 of BTV-1 was exchanged with S10 of BTV-8, BTV1/8NS3 by RG system (Fig. 3A). In addition, two other reassortants BTV1/8VP2.NS3 and BTV1/8VP5.NS3 were generated to determine the contribution of each segment in the context of NS3. Further, a sequence comparison between the segment S10 of BTV-1 and BTV-8 showed that only 12 residues in the NS3 sequence are different between these two serotypes, of which 5 are located at the putative extracellular domain of NS3. To determine if these differences play any role in pathogenicity, we introduced the substitutions L_149_I/S_153_K/A_154_T/I_156_V/Q_158_S in the BTV-1 NS3 sequence and generated a mutant virus BTV1/NS3_IKTVS_ that expressed an NS3 protein containing an extracellular domain similar to that of BTV-8. Each reassortant virus was grown in BSR cells and stocks were stored at 4 °C. Although all viruses yielded titers between 1.5 × 10^7^ and 4.6 × 10^7^ PFU/ml, BTV1/8VP5.NS3 yielded consistently lower titers (∼1 × 10^6^) than the other reassortant viruses.

Further, the growth of each virus was examined in ovine SFT-R cells. Interestingly, both BTV1/8NS3 and BTV1/NS3_IKTVS_ single reassortant viruses behaved like BTV-1, not BTV-8. The other two dual reassortant viruses, BTV1/8VP2.NS3 and BTV1/8VP5.NS3, behaved as an intermediate between two parental viruses as shown in Fig. 3B, suggesting that NS3 might be acting in concert with each of the outer capsid proteins.

**Fig. 4:**
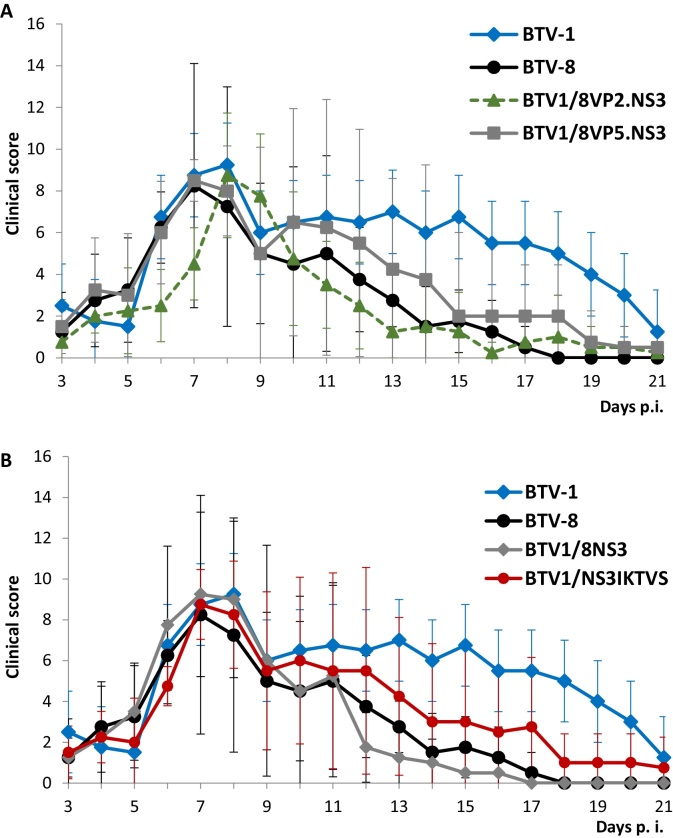
Pathogenicity of reassortants BTV1/8VP2.NS3, BTV1/8VP5.NS3, BTV1/8NS3 or mutant BTV1/NS3_IKTVS_ viruses. (A, B) Clinical signs were recorded for sheep infected with BTV1/8VP2.NS3 (A), BTV1/8VP5.NS3 (A), BTV1/8NS3 (B) or mutant BTV1/NS3_IKTVS_ (B) viruses using the same scoring described in Fig. 2. Parental BTV-1 and BTV-8 viruses were also included as control (same set of data was plotted in each graph).

For pathogenicity studies, animals were divided into six infection groups and a control group with four sheep in each group. Sheep in the infection groups were inoculated with BTV-1 or BTV-8 or one of four different reassortant BTV-1/BTV-8 viruses. Animals were clinically scored daily and blood samples were taken before and at days 1–11, 14, 17, and 21 days after infection. All the generated viruses (reassortants and mutant) were able to replicate in sheep, as real-time RT-PCR demonstrated an increasing amount of S6 RNA in blood samples over time (data not shown). When the animals were clinically scored, similar to the parental BTV-1, a delay in pathogenicity was observed for the virus BTV1/8VP2.NS3 with a maximum score at 8 days p.i. (Fig. 3B). The reassortants BTV1/8VP5.NS3, BTV1/8NS3, mutant BTV1/NS3I_KTVS_ and BTV-8 reached a maximum score at seven days p.i. (Fig. 4A and B). The clinical scores in all infection groups were significantly different from the control group; however, the observed differences between the infection groups were not statistically significant.

## 4 Discussion

BTV serotypes, BTV-1 and BTV-8, have displayed a marked ability to spread and severe virulence has been reported during European outbreaks in domestic ruminants, sheep being among the most severely affected livestock species (Allepuz et al., 2010, Elbers et al., 2008b). Detailed investigations in sheep to elucidate the pathogenic mechanisms of BTV-1 under experimental conditions are scarce (Hamblin et al., 1998, Perez de Diego et al., 2011). In comparison, infections carried out with BTV-8 in sheep have been accompanied by a significant variation in results (Darpel et al., 2007, Elbers et al., 2008c, Worwa et al., 2010). Recently, two studies have also reported a comparative study involving pathogenicity of BTV-1 and -8 in sheep and cattle (Dal Pozzo et al., 2013, Sanchez-Cordon et al., 2013). This is the first study to investigate how the difference in the clinical manifestations between BTV-1 and BTV-8 is influenced by the genetic origin of the virus. Since there is some evidence in previous reports regarding the involvement of VP2 and VP5, the two most exposed viral capsid proteins as determinants of virus virulence (DeMaula et al., 2000, Huismans and Howell, 1973), we have explored whether variations in pathogenicity are likely to be associated with the two most variable BTV genomic segments, S2 and S6 that encode VP2 and VP5 respectively. In addition, as S10, the gene encoding for NS3 has also been found to be the most variable amongst the segments encoding for the non-structural proteins in BTV infected cells, this protein was also hypothesized to play a potentially important role in BTV pathogenesis. Interestingly, in BTV infected cells it has also been demonstrated that the interaction of VP2 and VP5 with NS3 has an important role in virus assembly and trafficking (Beaton et al., 2002, Bhattacharya and Roy, 2008, Celma and Roy, 2009, Celma and Roy, 2011, Wirblich et al., 2006).

For this purpose, the BTV RG system was utilized to generate reassortant viruses that consisted of a backbone of BTV-1, with VP2, VP5 and NS3 of BTV-8 in five different combinations (BTV1/8VP2.5, BTV1/8VP2.5.NS3, BTV1/8VP2.NS3, BTV1/8VP5.NS3 and BTV1/8NS3). In each of the reassortants the equivalent genome segment of BTV-1 was replaced by BTV-8. This is the first time that reassortant viruses generated by RG technology have been tested in an animal host.

In order to investigate the influence of tissue specificity in the growth pattern of the reassortant viruses and to compare it with the two parental strains of BTV-1 and BTV-8, sheep kidney (PT) and thymus (SFT-R) cell lines were infected with the respective viruses. Since our primary objective was to assess the importance of the VP2, VP5 and NS3 in BTV pathogenesis, growth curves and virus protein production of reassortant virus BTV1/8VP2.5.NS3 were assessed in the two different ovine cell types. Our results showed that both wild-type viruses as well as the reassortant viruses had different growth profiles in each cell line, indicating that there may be a tissue specificity related to a particular strain that may play a role in virus pathogenicity. In addition, our results also revealed that the growth pattern of BTV1/8VP2.5 and not BTV1/8VP2.5.NS3 was similar to that of BTV-1 in the ovine kidney cell line. Experiments undertaken with BTV1/8VP2.5.NS3, BTV-1 and BTV-8 in ovine thymus cell lines demonstrated a more pronounced similarity in growth profile between BTV1/8VP2.5.NS3 and BTV-8 but not BTV-1. This confirmed that substituting all 3 segments, S2, S6 and S10 of BTV-1, with that of BTV-8 resulted in a reassortant virus that was more similar in behavior to BTV-8 than BTV-1. Since the effect was more pronounced in the ovine thymus SFT-R cell line, this also validated that in cell culture systems the origin of the cultured cells does play a significant role in determining virus growth characteristics. Hence, on the basis of these studies it was postulated that the growth profile of a particular BTV strain was dependent on VP2, VP5 and NS3.

Consequently, experiments designed to analyze the pathogenicity of BTV1/8VP2.5 and BTV1/8VP2.5.NS3 in sheep revealed that parental BTV-8 and reassortant BTV1/8VP2.5.NS3 triggered an earlier onset of clinical disease symptoms than parental BTV-1 and reassortant BTV1/8VP2.5. Interestingly, our results are in direct contrast to another recent investigation undertaken in sheep that have demonstrated that BTV-1 is more pathogenic than BTV-8. The difference in pathogenicity of BTV-1 and BTV-8 between our current study and that undertaken by Sanchez-Cordon et al. (2013) can be attributed to the fact that the BTV-1 strain used to recover our reassortant viruses was a tissue culture adapted strain obtained from South Africa. Subsequently, RT-PCR on blood samples procured from infected sheep and serum neutralization tests also confirmed that BTV1/8VP2.5 and BTV1/8VP2.5.NS3 were able to replicate in animals and that they also were able to generate a strong immune response similar to that of the parental strains. Since sheep infected with reassortant BTV1/8VP2.5.NS3 showed similar clinical scores to that of BTV-8, it led us to further hypothesize that of the 3 proteins, VP2, VP5 and NS3, the non-structural protein NS3 might be an important contributing factor for the development of BT disease. Hence, three more reassortant viruses were generated by RG system in which S10 of BTV-8 was either present singly (BTV1/8NS3) or in a combination with either BTV-8 VP2 (BTV1/8VP2.NS3) or VP5 (BTV1/8VP5.NS3). Sequence comparison of S10 belonging to BTV-1 and BTV-8 highlighted the presence of only 12 amino acid residues that were variable between the two serotypes. Since a cluster containing 5 of these variable residues was located in the putative extracellular domain of NS3, a mutant virus was generated (BTV1/NS3_IKTVS_) that only contained amino acid substitutions (L_149_I/S_153_K/A_154_T/I_156_V/Q_158_S) in this region of NS3. Consequently, this domain of BTV-1 NS3 became identical to the extracellular domain of BTV-8. When the reassortant and the mutant virus were inoculated in sheep, real-time RT-PCR of blood samples demonstrated that all the newly generated viruses and the two parental strains could replicate in sheep. Although pathogenicity analysis of the reassortant viruses suggested that BTV1/8VP2.NS3 behaved similarly to BTV-1, while the other two reassortant viruses (BTV1/8NS3 and BTV1/8VP5.NS3) and the mutant virus BTV1/NS3_IKTVS_ were similar to BTV-8, the overall clinical scores were not significantly different. Based on our result it was hypothesized that the two outer capsid proteins (VP2 and VP5) and the non-structural protein NS3 are all acting in concert with each other and are to a certain extent responsible for the pathogenicity of a particular strain. Interestingly, earlier reports have demonstrated that the interaction of NS3 with the two outer capsid proteins and cytoplasmic proteins have important consequences for both virus assembly and trafficking in infected cells (Beaton et al., 2002, Bhattacharya and Roy, 2008, Celma and Roy, 2009, Celma and Roy, 2011, Wirblich et al., 2006). Since mutant virus BTV1/NS3_IKTVS_, consisting of an extracellular loop region identical to that of BTV-8 NS3, did not show a statistical significant difference in virus pathogenicity when compared to the two parental strains, it can be concluded that the loop region of NS3 or its interaction with various cellular partners does not have a significant contribution to BT pathogenesis. Although the role of the remaining seven highly conserved BTV genomic segments cannot be negated, further work on this aspect is beyond the remit of this paper. Clinical experiments undertaken with BTV-1 and BTV-8 showed a different profile in two different sets of experiments (Fig. 2, Fig. 4). Since these two animal experiments were conducted over one year apart, it is possible that in spite of every effort that was made to keep conditions consistent between experiments, some variations occurred. However, it is unlikely that the small variation between experiments invalidates the findings made within each of the experiments. The role of NS3 in regulating interferon response has recently been reported (Chauveau et al., 2013). It will be interesting to investigate interferon responses of the triple reassortant virus versus the two wild-type viruses in the animal. However, these will require a series of experiments and detailed analysis that are beyond the remit of this study.

## 5 Conclusion

The clinical disease and pathogenicity of BTV is a very complex process and cannot be explained by the presence or absence of only one genome segment or its protein product. Much more work needs to be undertaken to clearly understand the genetic basis of BT pathogenesis and to our knowledge, this is the first report in this particular research area.
